# On the Therapeutic Properties of Carbolic Acid

**Published:** 1864-01

**Authors:** Crace Calvert


### Selections.

ON THE THERAPEUTIC PROPERTIES OF CARBOLIC ACID.—*By Crace Calvert*, PH. D., F. R. S.—I deem it my duty to draw the attention of the medical profession to the valuable therapeutic properties of carbolic acid, which I have during the last two years brought under the notice of some of the leading medical practitioners of Manchester and London. Before giving particulars of the chief applications of this substance which have been made by these gentlemen, I will first state what carbolic acid really is.

Carbolic acid, hydrated oxide of phenyle, or phenic acid, is a white substance, which crystallizes in long prisms, fusible at 93° Fah., and boiling at 370°. It has a slight tarry and aromatic smell, resembling that of wood creosote, and is freely soluble in alcohol, ether, and glycerine, particularly so in glacial acetic acid, and only slightly so in water, of which 100 parts will dissolve only three parts of carbolic acid It is easily prepared by treating the oils of tar, which distil between 350° and 400°, with caustic lye, removing the caustic lye solution from the neutral oils, and adding hydrochloric acid to the alkaline solution, when the carbolic acid is liberated, and rises to the surface as an oily fluid, from which, by distillation, the abovementioned therapeutic agent is obtained.

My friend and colleague, Thomas Turner, Esq., F. R. C. S., and Senior Surgeon at the Manchester Royal Infirmary, read, at the last meeting of the Lancashire Branch of the British Medical Association, a lengthy paper "On the uses of carbolic acid as a remedial agent," from which I extract the following:

"In cases of relaxation of the mucous surfaces, the solution of carbolic acid in glycerine, applied by means of a brush or sponge, is most beneficial. Thus its use is indicated in polypi of the nostrils, as well as ozæna, and in all putrid discharges from the mouth, throat, nostrils, ears, rectum, and vagina.

"I shall next call your attention to the use of carbolic acid in *Diptheria*, in which disease it is a most valuable remedy used topically to the throat. * * * To apply it I use a sponge mop, which should be used freely, but not saturated, lest a drop should fall into the larynx. The escharotic effect of carbolic acid is confined to the surface to which it is applied, there being no spreading to the contiguous parts, which may happen in the case of nitric acid. The aqueous solution of carbolic acid may be also used as a gargle.

"*Ulcers.*—I apply carbolic acid in different degrees of solution, according to the character of the sore, to carbuncle and ill-conditioned sores."

Mr. Turner also, in a private note to me, speaks of the use of carbolic acid to fœtid ulcers, in the following terms:— "It may be advantageously used, as a solution of one part of acid in forty parts of water, in fœtid ill-conditioned ulcers. It alters the action of the blood-vessels, causing a purulent instead of a sanious discharge, and destroys almost immediately the offensive smell of the secretion. In ulcers having a communication with carious bone, or even necrosis (where the bone is dead), it has, in its diluted state, a good effect when injected into the sinuses leading to the diseased bones. When there is mere caries or ulceration of the bone, it effects the healing process, and in necrosis it promotes the exfoliation of the dead portion. In gangrenous and all offensive sores, it removes all disagreeable smell and putrescency, and may render the discharge innocuous to the contiguous living and unaffected tissues. In its diluted state, therefore, it is a great boon to patients laboring under that class of disease."

When Mr. Turner wishes to employ carbolic acid in a less diluted state than the aqueous solution, and yet not in its full strength as a caustic, he prefers the following solution: He mixes two drachms of pure corbolic acid in one drachm of liquor potassæ and half a pint of water.

It is with pleasure that I am able to add that Mr. Oscar

Clayton, F.R.C.S., and Mr. Campbell De Morgan, F.R.C.S., have informed me of several successful applications which they have made of carbolic acid, confirming many of the results of Mr. Turner's, above described.

Mr. Oscar Clayton states that in two cases of fœtor of breath, arising from a diseased state of the mucous membrane covering the tonsils, he applied to the parts a mixture of equal proportions of glycerine and carbolic acid, and with perfect success.

Mr. Campbell De Morgan has also applied the glycerine solution of carbolic acid with success to several cases of lupus.

Dr. James Whitehead, of Manchester, prefers treating this disease (lupus) with an ointment made of half a drachm of carbolic acid to one ounce of spermaceti ointment.

Mr. Oscar Clayton has also successfully employed the aqueous solution to several skin diseases, to-wit: lepra, tinea capitis, rupia, etc.

Dr. Roberts and other medical men have employed carbolic acid with advantage internally for dyspepsia and other derangements of digestion.

Dr. Patterson, of St. Johns-wood, writes to me as follows: "I have prescribed your carbolic acid in several cases of fungoid disease during the last nine months with marked success. In three cases of fungus hæmatodes in which I employed it, the disease in all was checked in a remarkable manner. A thick crust was speedily formed on the ulcerated and bleeding surfaces, the exhausting discharges were completely arrested, and in one case there was great diminution in the size of the fungous mass. Your carbolic acid is almost a specific in cases of anthrax."

I also think it well to insert the following remarks from Dr. Thomas Hughes, M.R.C.S., F.S.A., London, August 29, 1863:—"Sir: I have used Dr. Calvert's carbolic acid as an external application in cases of sloughing wounds with the most marvellous effect; and in no case was its effect more strikingly manifest than in the case of Rogers, one of your miners, who received such a contusion of the hand as to destroy the arteries leading to the index and little fingers and, in spite of every effort made to restore the circulation in the fingers, sloughing took place, and which appeared to spread and extend to the hand and arm with such rapidity, that if it had not been for the timely application of the carbolic acid the man would have lost his arm from the most destructive sloughing I ever witnessed. The effect of carbolic acid was so decidedly marked as to leave no doubt of its wonderful effects in checking the spreading of sloughing, and in accelerating the separation of slough. It seemed also to have the effect of promoting the growth of granulations. and hastening the healing of the wounds. I have used carbolic acid in several other cases with the same happy effect."

Two eminent French physiologists, MM. Gratiolet and Lemaire, have published a most interesting paper on the action of carbolic acid in arresting putrefaction; and they have made the important observation that, while it does not interfere with chemical fermentations, such as the conversion of amygdaline into hydruret of benzoile, and the conversion of myronic acid by myrosyne, it completely arrests all vegetable and animal fermentations which arise from cryptogamic life. They have also observed that when carbolic acid is mixed with the vaccine virus, it entirely prevents its peculiar action upon animal organization.

These valuable observations of MM. Gratiolet and Lemaire strongly impress me with the idea that the use of carbolic acid might prove of great advantage in the early stages of consumption, if applied in the following manner, to-wit: by making the patient frequently inhale the vapor of the acid by means of an inhaler containing some cotton-wool saturated with the acid so that the inspired air must pass through the wool. I would at the same time administer a teaspoonful of the aqueous solution, mixed with two ounces of peppermint-water, three times a day. I think also that the same treatment might be advantageously tried in cases of scarlatina and typhoid fever, with the addition of saturating the air of the chamber as far as possible with the vapor, by placing lint of wool steeped in carbolic acid in various parts of the room. I would also administer once a day an enema consisting of a weak solution of carbolic acid.—*London Lancet*, Sept. 26.

---

Nitrate of Silver.—*By John Higginbottom*, F. R. S.—I think it very important to call the attention of surgeons to the superiority of ordinary nitrate of silver over the new preparations which have been now some time in use. The new preparation—"lunar caustic points, perfectly tough"—is worthless as an application in surgical cases. It is not

